# Bionics and Human Biomechanics Applied in Intelligent Crash Tests of Cars

**DOI:** 10.1155/2019/8750413

**Published:** 2019-01-08

**Authors:** Tao Xu, Tatsuo Yoshino, Shujun Zhang, Guowu Wei, Zhixin Liu

**Affiliations:** ^1^School of Mechanical Science and Engineering, Jilin University, Changchun, China; ^2^School of Computing and Technology, University of Gloucestershire, Cheltenham, UK; ^3^School of Computing, Science & Engineering, University of Salford, Salford, UK; ^4^China Automotive Technology and Research Center (CATARC), Tianjin, China

## 1. Introduction

Improving crash safety of cars has become an important content in the research of automotive safety. The optimum design of intelligent crash test technology and devices for automotive safety has become a hot issue in the field. In order to test active safety or passive safety by braking or crash tests, intelligent testing devices (also called anthropomorphic test devices (ATDs) or crash test dummies) need to describe and simulate the damage mechanism and the tolerance limit of biological characteristics of the human body, such as dynamics and kinematics response to impact or acceleration. This is essential to protect human beings from car accidents.

Along with the increasing exploration of nature, organisms with rigid flexible coupling structures are gradually discovered, which have excellent performances such as impact resistance, abrasion resistance, and drag reduction. The combination of bionics and biomechanics constantly brings about new inspiration and innovation to the field of engineering and automotive safety. So, this special issue called for original research articles on basic biomechanical researches of the human body, computer simulation for human body modeling and analysis, new development of intelligent anthropomorphic test devices for measuring the response of the human body in certain environments where an impact or other loadings are applied to the body, and the application of bionic structures in automobiles to improve their anticollision performance. The contents can involve bionics, biomechanics, automobile engineering, human body modeling, impact and contact mechanics, materials science of skin, and high-precision sensor informatics and mechanical processing technology. The highly integrated dummy design is also subject to various standard calibration tests, so this special issue is a cross-discipline.

## 2. Injury Criteria

In crash tests of cars, each part of the occupant could be injured to varying degrees. Some injury criteria of key parts are shown in Table 1, and the specific meanings of the symbols can be found in [1].

## 3. Description of the Special Issue

This special issue accepted 5 papers out of 10 after careful reviewing by editors, which leads to an acceptance ratio of 50%. The 5 papers focuse on crash dummy improvement, injury characteristics of knee joints, and injury in some special conditions.

T. Xu et al. review the development and validation of dummies and human models used in crash tests. The mechanical dummies are introduced according to the collision types: frontal impact dummy, side impact dummy, and rear impact dummy. And the human model section details WSU, HUMOS, THUMS, and GHBMC human models. From the article, we can see the technological progress of the dummies and the human body models. Injury criteria and biomechanical tests in early time are also introduced.

Y. Xiong et al. explore the mechanical response and injury characteristics of knee joints at different speeds using impact experiment with cadaveric knee samples and finite element simulation. The tests are all carried under conditions of longitudinal impacts, and the results all show that low-speed impact mainly leads to medial injuries, while high-speed impact leads to both medial and lateral injuries. The study can provide research basis for the prevention and treatment of longitudinal impact injuries of knee joints.

S. Wang et al. use finite element models to analyze how the panel design parameters can affect occupant head injuries. The paper focuses on the three factors of panel hardness, elastic modulus of the filling and frame, and the distance from fixed location. The findings indicate that a soft panel with a long fixing distance is beneficial for the head prevention.

I. L. Cruz-Jaramillo et al. study the head injury criterion (HIC) and chest severity index (CSI) with a 6-year-old Hybrid III dummy in the low-back booster (LBB) passive safety system. The findings of this study suggest that using materials, the attachment system of the LBB and the belt restraint system properly placed over the infant trunk are the main factors to reduce the injury criterion rate.

H. Guo et al. applied a novel biobjective algorithm Newton Neumann Series Expansion Frisch Algorithm (NNSEFA) to dummy head FE experiment. By optimizing the dummy head with the algorithm, the improved model has a better accuracy in the collision simulation. The application of the biobjective optimization algorithm provides new ideas for occupant safety design.

## Figures and Tables

**Table 1:** Injury criteria of key parts.

Part	Injury criterion	Formula
Head	HIC	HIC = (t_2_ − t_1_)[1/t_2_ − t_1_∫_*t*_1__^*t*_2_^*adt*]^2.5^
Neck	*N*_*ij*_	*N*_*ij*_ = (*F*_*z*_/*F*_int_) + (*M*_*y*_/*M*_int_)
Chest	CTI	CTI = (*A*_max_/*A*_int_) + (*D*_max_/*D*_int_)
Tibia	TI	TI = (*M*_*R*_/*M*_*R*max_) + (*F*_*Z*_/*F*_*Z*max_)
